# Breast cancer cells obtain an osteomimetic feature *via* epithelial-mesenchymal transition that have undergone BMP2/RUNX2 signaling pathway induction

**DOI:** 10.18632/oncotarget.12939

**Published:** 2016-10-27

**Authors:** Cong-Cong Tan, Gui-Xi Li, Li-Duan Tan, Xin Du, Xiao-Qing Li, Rui He, Qing-Shan Wang, Yu-Mei Feng

**Affiliations:** ^1^ Department of Biochemistry and Molecular Biology, Tianjin Medical University Cancer Institute and Hospital, National Clinical Research Center of Cancer, Tianjin 300060, China; ^2^ Key Laboratory of Breast Cancer Prevention and Treatment of the Ministry of Education, Tianjin Medical University Cancer Institute and Hospital, National Clinical Research Center of Cancer, Tianjin 300060, China

**Keywords:** osteomimicry, epithelial-mesenchymal transition, BMP2/RUNX2 signaling pathway, breast cancer bone metastasis, cancer-associated fibroblast

## Abstract

Bone is one of the most common organs of breast cancer metastasis. Cancer cells that mimic osteoblasts by expressing bone matrix proteins and factors have a higher likelihood of metastasizing to bone. However, the molecular mechanisms of osteomimicry formation of cancer cells remain undefined. Herein, we identified a set of bone-related genes (BRGs) that are ectopically co-expressed in primary breast cancer tissues and determined that osteomimetic feature is obtained due to the osteoblast-like transformation of epithelial breast cancer cells that have undergone epithelial-mesenchymal transition (EMT) followed by bone morphogenetic protein-2 (BMP2) stimulation. Furthermore, we demonstrated that breast cancer cells that transformed into osteoblast-like cells with high expression of BRGs showed enhanced chemotaxis, adhesion, proliferation and multidrug resistance in an osteoblast-mimic bone microenvironment *in vitro*. During these processes, runt-related transcription factor 2 (RUNX2) functioned as a master mediator by suppressing or activating the transcription of BRGs that underlie the dynamic antagonism between the TGF-β/SMAD and BMP/SMAD signaling pathways in breast cancer cells. Our findings suggest a novel mechanism of osteomimicry formation that arises in primary breast tumors, which may explain the propensity of breast cancer to metastasize to the skeleton and contribute to potential strategies for predicting and targeting breast cancer bone metastasis and multidrug resistance.

## INTRODUCTION

Breast cancer commonly metastasizes to the bone in women with advanced disease, which can cause debilitating skeletal complications, such as bone destruction and associated bone pain, fracture, hypercalcemia, and paralysis due to spinal cord compression [1]. The affinity of cancer cell metastases for bone relies on intrinsic biological properties [2] and specific interactions with the bone microenvironment [3]. Breast cancer cells can acquire an osteoblast-like phenotype by ectopically expressing bone matrix proteins (e.g., bone sialoprotein (BSP) [4], osteopontin (OPN) [5], osteoprotegerin (OPG) [6] and secreted protein acidic and cysteine rich (SPARC)/osteonectin (ON) [7]), osteoblast-specific cadherins (e.g., cadherin 11 (CDH11) [8]), transcription factors that regulate bone remodeling (e.g., runt-related transcription factor 2 (RUNX2) [9]) and an “osteoblast gene signature” [2]. Cancer cells that highly express these bone-related genes (BRGs) preferentially home to [6], colonize in [8, 10], and survive in [11] bone. However, the clinical relevance of BRGs to bone-specific metastasis and how osteomimetic properties emerge in primary tumors remain poorly understood.

Cancer-associated fibroblasts (CAFs), which are activated by tumor cells, are the predominant type of stromal cell in breast cancer tissues. CAFs reciprocally promote aggressive phenotypes of breast cancer cells through paracrine signals (e.g., TGF-β) that trigger epithelial-to-mesenchymal transition (EMT) [12]. EMT is an important mechanism that enables cancer cells to complete various steps in the metastatic cascade by transiting to a mesenchymal cell phenotype [13] with stem cell-like properties [14]. Epithelial cancer cells that have undergone EMT exhibit multi-lineage differentiation potential similar to mesenchymal stem cells and can differentiate into myofibroblasts/CAFs [15], osteoblasts, chondrocytes and adipocytes [16] in response to tissue-specific differentiation signals. Bone morphogenetic proteins (BMPs) are key osteogenic factors that induce the maturation of mesenchymal cells into osteoblasts by activating various osteogenesis-related transcription factors, such as RUNX2 [17]. BMP2 has been reported to be expressed in breast cancer cell lines [18] and primary breast cancer tissues [19], and it is particularly elevated in breast cancer bone metastatic samples relative to metastases in other organs [20]. These pieces of evidence led us to hypothesize that the osteomimetic phenotype of breast cancer cells that ectopically co-express BRGs may be derived from EMT followed by the induction of osteogenic differentiation signals in the tumor microenvironment, which mimics the process of osteoblastic differentiation under normal physiological conditions.

In the current study, we provided clinical evidence to reveal that breast cancers present an osteomimic feature with the ectopic co-expression of a set of BRGs which preferentially metastasize to bone. Mechanistically, we demonstrated that the ectopic co-expression of BRGs in breast cancer cells is derived from EMT that has undergone BMP2 induction. Functionally, we validated the propensity of osteomimetic breast cancer cells for chemotaxis, adhesion, proliferation and multidrug resistance in an osteoblast-mimic bone microenvironment *in vitro*. Furthermore, we demonstrated that RUNX2 serves as a master mediator of the formation of an osteomimetic phenotype by activating the transcription of BRGs in breast cancer cells.

## RESULTS

### Breast cancers present an osteomimetic feature with the ectopic co-expression of a set of BRGs which has a tendency to metastasize to bone

**Figure 1:**
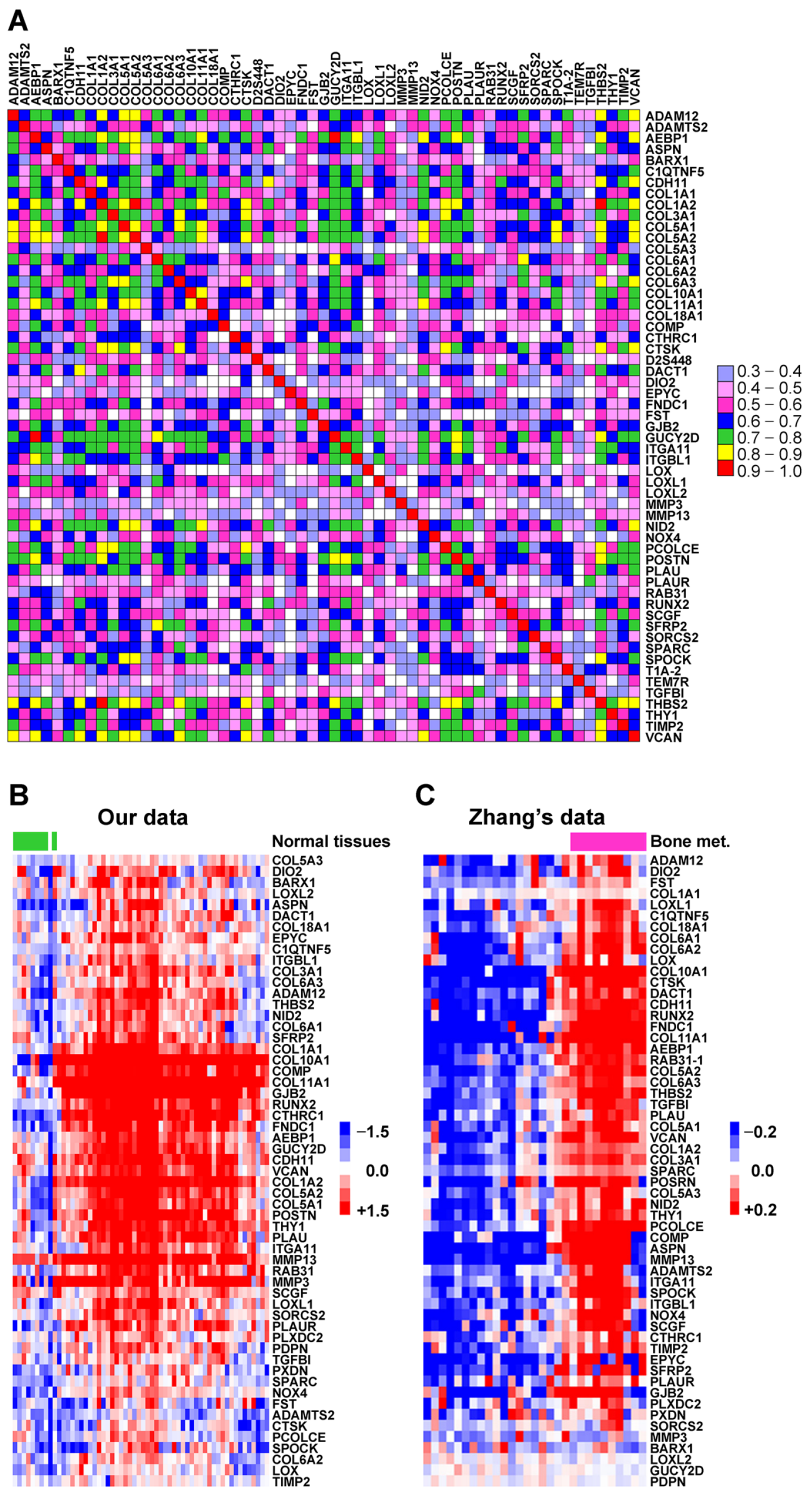
Breast cancers present an osteomimetic feature with the ectopic co-expression of a set of BRGs which are prone to bone metastasis **A.** Schematic diagram of Pearson's correlation coefficients for the mRNA expression of 57 BRGs between each other, based on our gene expression profiling dataset of 49 primary breast cancer tissues. Correlation coefficient that greater than 0.3 is defined as a significant correlation. The color box spectrum represents different Pearson's correlation coefficients between corresponding genes. **B.** Comparison of mRNA expression of the set of co-expressed BRGs between normal breast tissues and breast cancer tissues. The data were mined from our gene expression profiling datasets of 9 human normal breast tissues and 49 primary breast cancer tissues. The heat map depicts the mRNA expression levels of each gene in different samples. The green bars indicate normal breast tissues, and the others are breast cancer tissues. **C.** Comparison of the mRNA expression of the set of co-expressed BRGs in bone metastatic breast cancer tissues and metastatic breast cancer tissues from other organ. The data were mined from Zhang's gene expression profiling dataset of 29 metastatic breast cancer tissues (GSE14017). Heat map depicts the mRNA expression levels of each gene in different samples. The purple bars indicate bone metastatic samples, and the others are metastatic samples from other organs.

To investigate the intrinsic features of breast cancer, we analyzed the co-expression of genes in breast cancer tissues based on our gene expression profiling dataset of 49 primary breast cancer tissues [21] using Pearson's correlation analysis. A total of 57 BRGs were identified as co-expressed genes with a correlation coefficient greater than 0.3 in breast cancer tissues, including genes that encode bone matrix proteins (ASPN, COMP, CSPG2/VCAN, DSPG3/EPYC, POSTN, SPARC, SPOCK, COLs), extracellular matrix (ECM)-degrading enzymes (MMPs, ADAMs, ADAMTSs, CTSK, PLAU/uPA) and their inhibitors (TIMPs), osteoblast cadherin (OB-cadherin/CDH11), osteoblast transcription factors (RUNX2), and cytokines (SCGF; Figure 1A). We then compared the mRNA levels of the set of BRGs between normal breast tissues (n = 9) and breast cancer tissues (n = 49) based on our gene expression profiling dataset [21]. The result revealed that these BRGs were more highly expressed in breast cancer tissues than in normal breast tissues (Figure 1B and Supplementary Figures S1A-S1C). To investigate whether the ectopically co-expressed BRGs contribute to breast cancer bone metastasis, we analyzed the expression of the set of BRGs in bone metastatic breast cancer tissues (n = 10) and metastatic breast cancer tissues in other organs (n = 19) based on Zhang's gene expression profiling dataset (GEO accession number: GSE14017) [20] (Figure 1C and Supplementary Figures S1D-S1F). The result showed that the set of BRGs was highly co-expressed in metastases in bone compared with those in other organs. Collectively, these findings indicate that breast cancers present an osteomimetic feature with ectopically co-expressed a set of BRGs, which have a tendency to metastasize to bone.

### The co-expression of BRGs in breast cancer cells is derived from EMT that has undergone BMP2 induction

**Figure 2:**
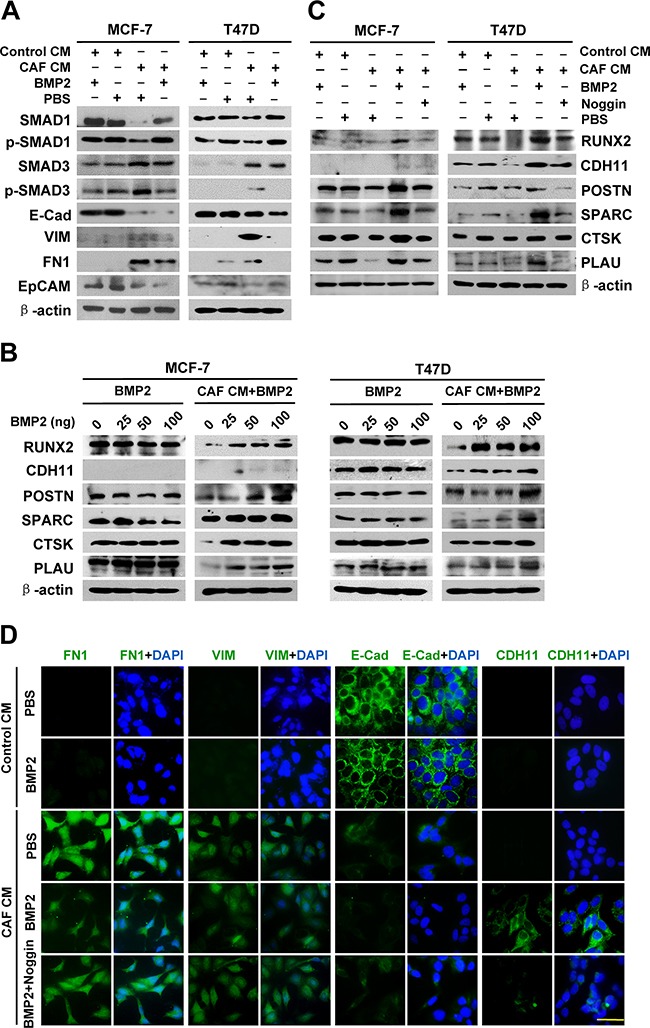
The co-expression of BRGs in breast cancer cells is derived from EMT that has undergone BMP2 induction MCF-7 and T47D cells were treated as indicated. **A.** The protein expression of SMAD1, p-SMAD1, SMAD3 and p-SMAD3, as well as EMT markers in the cancer cells was detected by immunoblot. β-actin was used as a loading control. **B.** and **C.** The protein expression of BRGs in cancer cells was detected by immunoblot. β-actin was used as a loading control. **D.** The protein expression and localization of FN1, VIM, E-Cad, and CDH11 (green) in MCF-7 cells was detected by immunofluorescence. DAPI staining (blue) indicates the nucleus. Scale bar, 40 μm.

Physiologically, BRGs are initially expressed in the process of directed differentiation of bone marrow mesenchymal stem cells under the induction of growth factors during bone development and the repair of bone damage. BMP2 is a pivotal signal in the induction of bone development and bone remodeling [22] by triggering the expression of skeletal-restricted genes, such as RUNX2, BSP, OPN, and OC, to drive the differentiation of mesenchymal cells [17]. Based on the facts that CAFs are enriched in the breast cancer microenvironment and can induce EMT in breast cancer cells [12] and that BMP2 is elevated in primary breast cancer tissues [19], we hypothesized that the ectopically co-expressed BRGs may be derived from epithelial cancer cells that have undergone EMT and acquired cancer stem cells (CSCs) properties, followed by BMP2 induction in the tumor microenvironment. To address this hypothesis, we treated epithelial breast cancer cells MCF-7 and T47D with the conditional medium from CAFs (CAF CM) to induce EMT as previously described [12] and then added BMP2 to induce the cells that had undergone EMT to transform into an osteoblast-like phenotype. The results showed that the p-SMAD3 was dramatically increased and p-SMAD1 was decreased by CAF CM induction, indicating the activation of the TGF-β/SMAD signaling pathway along with the suppression of the BMP/SMAD signaling pathway. Consistently, we observed EMT phenotype in the CAF CM-treated cells: the epithelial markers E-cadherin (E-Cad) and EpCAM were downregulated, and the mesenchymal markers fibronectin 1 (FN1) and vimentin (VIM) were upregulated. When the cells that had undergone EMT in response to CAF CM were further induced by BMP2, p-SMAD1 was significantly increased and p-SMAD3 was decreased, indicating that the BMP/SMAD signaling pathway was activated along with the suppression of TGF-β/SMAD signaling pathway (Figure 2A and Supplementary Figure S2A). Consistently, we observed increased expression of the bone matrix-remodeling proteins POSTN, SPARC, CTSK, and PLAU/uPA, as well as osteoblast cadherin CDH11 and transcription factor RUNX2 (Figure 2B and Supplementary Figure S2B). However, BMP2 alone had no such effect in either MCF-7 or T47D cells (Figures 2A and 2B and Supplementary Figures S2A and S2B).

To further validate the role of the EMT-BMP2 pathway in the induction of BRGs, we evaluated the expression of bone matrix-remodeling proteins in MCF-7 and T47D cells treated with CAF CM followed by adding BMP2 plus noggin. The results showed that the elevated expression of RUNX2, CDH11, POSTN, SPARC, CTSK, and PLAU/uPA in CAF CM/BMP2-treated MCF-7 and T47D cells was reversed by noggin (Figure 2C and Supplementary Figure S2C). Immunofluorescence staining revealed that epithelial cadherin (E-Cad) was converted into osteoblastic cadherin (CDH11) by CAF/BMP2 induction; however, when the BMP signaling pathway was blocked by noggin, the induction of CDH11 was attenuated (Figure 2D). These results suggest that BMP2 is essential for the induction of BRGs expression in breast cancer cells that have undergone EMT. Taken together, the above evidence demonstrates that mutual antagonism between the TGF-β/SMAD and BMP/SMAD signaling pathways regulates the transition of cells from differentiated epithelium to osteomimcry, in which the cells go through the processes of EMT and mesenchymal-osteomimetic transition (MOT).

### Epithelial cancer cells with ectopic co-expression of BRGs inducted by CAF/BMP2 gain the advantages of homing to, residing in and growing in the bone microenvironment

**Figure 3:**
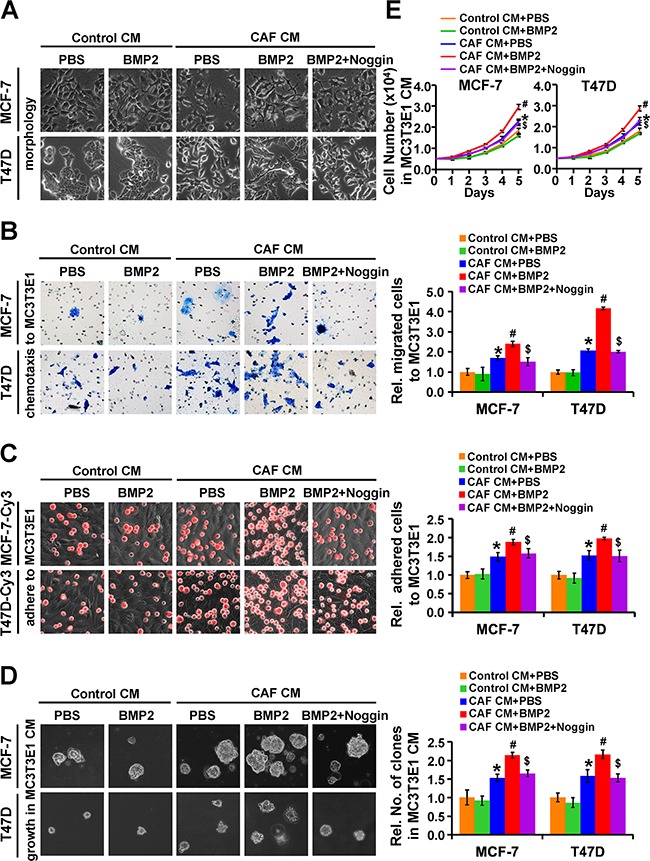
Epithelial cancer cells with co-expression of BRGs induced by CAF/BMP2 signaling gain the advantages of homing to, residing in and growing in an osteoblast-mimic bone microenvironment MCF-7 and T47D cells were treated as indicated. **A.** The morphology of the cancer cells. **B.** The chemotactic migration of cancer cells towards MC3T3E1 cells was assessed by transwell assay. **C.** The adhesion of cancer cells (red, labeled with Cy3) to MC3T3E1 cells was assessed by putting cancer cells on top of MC3T3E1 cells at 100% confluence and incubating the co-culture for 30 min. **D.** The colony formation of cancer cells in soft agar containing MC3T3E1 CM. Magnification: 200X. **E.** The proliferation of cancer cells in MC3T3E1 CM. Data are presented as the mean ± S.D. of three independent experiments performed in duplicate. *, *P* < 0.05 compared with Control CM plus PBS; #, *P* < 0.05 compared with cells treated with CAF CM plus PBS; $, *P* < 0.05 compared with cells treated with CAF CM plus BMP2.

Since the osteoblast-like phenotype enables cancer cells to reside and thrive in bone [2], we further investigated whether the ectopic co-expression of BRGs in response to CAF/BMP2 induction affects the abilities of chemotaxis, adhesion, anchorage-independent growth and proliferation of epithelial cancer cells in the bone microenvironment. We used MC3T3E1 preosteoblastic cells undergone induction of osteoblast differentiation and MG-63 osteosarcoma cells or their CM to mimic the bone microenvironment *in vitro* to examine the chemotactic migration of MCF-7 and T47D cells towards MC3T3E1 or MG-63 cells in co-culture, the heterogeneity adhesion of cancer cells to MC3T3E1 or MG-63 cells, and the soft agar colony formation and proliferation of cancer cells in MC3T3E1 or MG-63 CM. The results showed that both MCF-7 and T47D cells cultured with CAF CM showed an EMT phenotype, with a spindle-like shape and cell scattering, while the cells changed to exhibit a polygonal-shaped osteoblast-like morphology with thick pseudopodia after further BMP2 induction (Figure 3A). The chemotactic migration of MCF-7 and T47D cells towards MC3T3E1 or MG-63 cells (Figure 3B and Supplementary Figure S3A), heterogeneity adhesion to MC3T3E1 or MG-63 cells (Figure 3C and Supplementary Figure S3B), anchorage-independent growth in soft agar (Figure 3D and Supplementary Figure S3C) and proliferation (Figure 3E) in MC3T3E1 or MG-63 CM were significantly increased after CAF CM/BMP2 induction, while these enhanced capabilities were attenuated by noggin. However, BMP2 induction alone did not alter these behaviors (Figures 3A-3E and Supplementary Figures S3A-S3C). These results indicate that epithelial cancer cells with ectopic co-expression of BRGs that were derived from EMT undergone BMP2 induction acquire the capabilities to home to, reside in and grow in the bone microenvironment.

### RUNX2 is a critical mediator for CAF/BMP2-induced expression of BRGs in breast cancer cells

**Figure 4:**
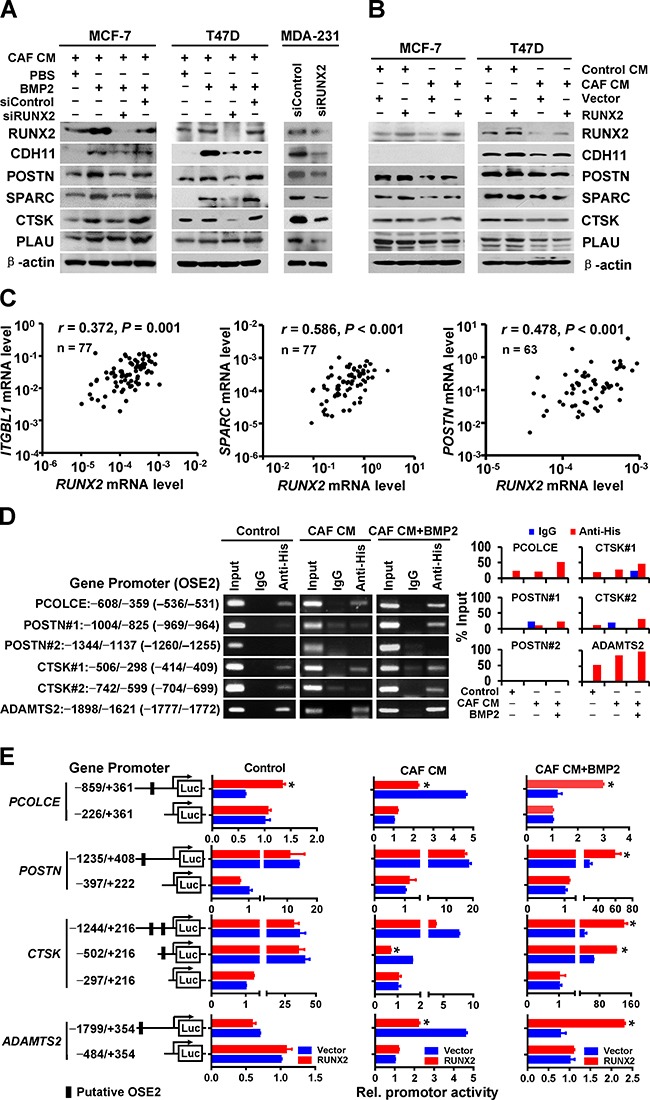
RUNX2 mediates the CAF/BMP2-induced co-expression of BRGs in breast cancer cells through directly and indirectly regulating their transcription **A.** The protein levels of BRGs in MCF-7, T47D and MDA-MB-231 cells treated as indicated were detected by immunoblot. β-actin was used as a loading control. For MCF-7 and T47D cells, RUNX2 was knocked down by RUNX2 siRNA (siRUNX2) transfection in the presence of 100 ng/mL BMP2 for 3 days after a 3-day culture with CAF CM. For MDA-MB-231 cells, RUNX2 was directly knocked down by siRUNX2 transfection. **B.** The protein levels of BRGs in CAF CM- or Control medium-cultured MCF-7 and T47D cells with the transfection of RUNX2 plasmids or vector control were detected by immunoblot. β-actin was used as a loading control. **C.** The correlation of *ITGBL1* (n = 77), *SPARC* (n = 77) or *POSTN* (n = 63) mRNA levels with *RUNX2* mRNA levels in primary breast cancer tissues was analyzed by Pearson's correlation analysis. The mRNA expression of each gene in primary breast cancer tissues was detected by RT-QPCR. **D.** The binding of RUNX2 to the proximal promoter regions of *PCOLCE*, *POSTN*, *CTSK*, and *ADAMTS2* containing or lacking OSE2 in MCF-7 cells transfected with pCDNA3.1-RUNX2-His was assessed by ChIP assay with an anti-His antibody. Isotype IgG was used as a negative control, and the total input was used as a positive control. **E.** The promoter activity of *PCOLCE*, *POSTN*, *CTSK* and *ADAMTS2* containing or lacking OSE2 in MCF-7 cells treated as indicated was detected by dual-luciferase reporter assay. Two independent experiments were performed in duplicate. The data are presented as the mean ± SD. *, *P* < 0.05 compared with cells transfected with the corresponding vector control.

RUNX2 is an essential transcription factor for osteoblast differentiation and bone remodeling by directly regulating multiple skeletal-restricted genes [23]. To investigate whether RUNX2 mediates the ectopic expression of BRGs during CAF/BMP2 induction in epithelial cancer cells, we knocked down RUNX2 expression in MCF-7 and T47D cells by RUNX2 siRNA (siRUNX2) transfection in the presence of BMP2 for 3 days after cultured with CAF CM for 3 days. We found that RUNX2 depletion resulted in significant down-regulation of the CAF/BMP2-induced expression of CDH11, POSTN, SPARC, CTSK, and PLAU/uPA compared with the control (Figure 4A and Supplementary Figure S4A). CDH11 expression was verified by immunofluorescence (Supplementary Figure S4B). Conversely, RUNX2 knockdown in MDA-MB-231 cells, a bone metastatic breast cancer cell line with high RUNX2 expression [24], down-regulated the expression of these BRGs (Figure 4A and Supplementary Figure S4A). However, the forced expression of RUNX2 in MCF-7 and T47D cells cultured with either Control CM or CAF CM did not significantly alter the protein expression levels of these BRGs, although SPARC changed a small amount in MCF-7 cells and CDH11 in T47D cells (Figure 4B and Supplementary Figure S4C). Moreover, we observed a positive correlation of the mRNA levels between *RUNX2* and BRGs (*ITGBL1*, *SPARC* and *POSTN*) in primary breast cancer tissues (Figure 4C). These results suggest that the ectopic co-expression of BRGs in breast cancer cells induced by CAF/BMP2 is mediated through the up-regulation of RUNX2, and the activation of the BMP/SMAD signaling pathway is required for the RUNX2-mediated expression of BRGs.

### RUNX2 triggers the transcription of a set of BRGs in epithelial breast cancer cells that have undergone CAF/BMP2 induction

Multiple known transcriptional targets of RUNX2 are included in the set of BRGs, including *COL1A1* [25], *COL10A1* [26], *MMP13* [27] and *ITGBL1* [28]. To identify other transcriptional targets of RUNX2 in the set of BRGs, we analyzed the 2 Kb upstream region of the promoters for the RUNX2 binding sequence “ACCACA” that known as an osteoblast-specific cis-acting element 2 (OSE2). In addition to the known targets of RUNX2, 38 genes contained one or more OSE2 (Table 1). Chromatin immunoprecipitation (ChIP) assays showed the direct bindings of RUNX2 to the OSE2-containing regions of the promoters of *PCOLCE* (−536/−531), *POSTN* (−969/−964, −1260/−1255), *CTSK* (−414/−409, −704/−699) and *ADAMTS2* (−1777/−1772) in MCF-7 cells with RUNX2-His plasmid transfection, and these bindings were significantly increased (*PCOLCE*, *CTSK*, *ADAMTS2*) or dramatically appeared (*POSTN*) by CAF/BMP2 induction, but did not affected by CAF CM alone (Figure 4D). Dual-luciferase assays showed that RUNX2 overexpression only enhanced *PCOLCE* promoter activation but not the other promoters in MCF-7 cells, whereas it dramatically activated the promoters of all four genes when the cells were induced with CAF/BMP2 (Figure 4E). Interestingly, the cells treated with CAF CM alone showed reduced promoter activity of *PCOLCE*, *CTSK* and *ADAMTS2* by RUNX2 overexpression (Figure 4E), although the binding of RUNX2 to the promoters of these genes was not significant changed compared with the control cells, indicating that RUNX2 transcriptionally repress its targets of BRGs under the induction of TGF-β/SMAD signaling pathway. The TGF-β/SMAD and BMP/SMAD signaling pathways play opposite roles in regulating the function of RUNX2 transcription factor during these processes: the TGF-β/SMAD signaling pathway induces the transcriptionally repressing function of RUNX2, and the BMP/SMAD signaling pathway induces the transcriptionally promoting function of RUNX2. Thus, RUNX2 functions as a master mediator during the transformation of epithelial breast cancer cells into ostomimetic cells under CAF/BMP2 induction, and the expression of BRGs was repressed in the TGF-β/SMAD/RUNX2 signaling pathway, but induced in the BMP/SMAD/RUNX2 signaling pathway.

**Table 1:** BRGs potentially targeted by RUNX2

Gene symbol	References sequence number	Gene description	Site of OSE2 relative to the TSS
*AEBP1*	NM_001129	AE binding protein 1	−1395/−1390
			−1517/−1512
*ADAM12*	NM_001288973	ADAM metallopeptidase domain 12	−1962/−1957
*ADAMTS2*	NM_014244	ADAM metallopeptidase with thrombospondin type 1 motif 2	−1777/−1772*
*BARX1*	NM_021570	BARX homeobox 1	−1561/−1556
*C1QTNF5*	NM_015645	C1q and tumor necrosis factor related protein 5	−1310/−1305
			−1912/−1907
*CDH11*	NM_001308392	Cadherin 11	−1807/−1802
*COL1A1*	NM_000088	Collagen, type I, alpha 1	−237/−232 [25]**
*COL1A2*	NM_000089	Collagen, type I, alpha 2	−604/−599
*COL5A2*	NM_000393	Collagen, type V, alpha 2	−1472/−1467
*COL5A3*	NM_015719	Collagen, type V, alpha 3	−538/−533
			−977/−972
*COL6A2*	NM_058174	Collagen type VI, alpha 2	−646/−641
			−932/−927
			−965/−960
			−986/−981
			−1023/−1018
			−1042/−1037
			−1085/−1080
			−1465/−1460
*COL6A3*	NM_004369	Collagen type VI alpha 3	−370/−365
			−761/−756
			−1299/−1294
*COL10A1*	NM_000493	Collagen, type X, alpha 1	−358/−353 [26]**
*COMP*	NM_000095	Cartilage oligomeric matrix protein	−71/−66
*CTHRC1*	NM_001256099	Collagen triple helix repeat containing 1	−26/−21
*CTSK*	NM_000396	Cathepsin K	−414/−409*
			−704/−699*
*DACT1*	NM_001079520	Dishevelled-binding antagonist of beta-catenin 1	−1257/−1252
			−1444/−1439
*DIO2*	NM_013989	Deiodinase, iodothyronine, type II	−532/−527
			−1750/−1745
*FBN1*	NM_000138	Fibrillin 1	−1186/−1181
			−1305/−1300
			−1968/−1963
*GJB2*	NM_004004	Gap junction protein beta 2	−366/−361
			−1732/−1727
*ITGBL1*	NM_004791	Integrin beta-like 1	−793/−788 [28]**
*LOX*	NM_002317	Lysyl oxidase	−1033/−1028
			−1048/−1043
*LOXL2*	NM_002318	Lysyl oxidase-like 2	−1135/−1130
			−1646/−1641
*MMP3*	NM_002422	Matrix metallopeptidase 3	−978/−973
*MMP13*	NM_002427	Matrix metallopeptidase 13	−143/−138 [27]**
			−136/−131 [27]**
*NOX4*	NM_001143837	NADPH Oxidase 4	−280/−275
			−1526/−1521
*OGN*	NM_014057	Osteoglycin	−1821/−1816
*PCOLCE*	NM_002593	Procollagen c-endopeptidase enhancer	−536/−531*
*PDPN*	NM_006474	Podoplanin	−170/−165
*PLAU*	NM_002658	Plasminogen activator	−1724/−1719
*PLXDC2*	NM_032812	Plexin domain containing 2	−723/−718
*POSTN*	NM_006475	Periostin	−969/−964*
			−1260/−1255^#^
*SFRP2*	NM_003013	Secreted frizzled-related protein 2	−1749/−1744
*SPARC*	NM_001309443	Secreted protein acidic and cysteine rich	−866/−861
			−1203/−1198
			−1795/−1790
			−1874/−1869
*SPOCK*	NM_004598	Sparc/osteonectin, cwcv and kazal-like domains proteoglycan	−346/−341
*TGFBI*	NM_000358	Transforming growth factor beta-induced	−1296/−1291
*THY1*	NM_001311162	Thy-1 cell surface antigen	−1751/−1746
*TIMP2*	NM_003255	TIMP metallopeptidase inhibitor 2	−588/−583

* Note:, identified binding site of RUNX2 in this study;

** , identified target of RUNX2 in the reference's report;

# , identified non-binding site of RUNX2 in this study.

### RUNX2 is critical for the CAF/BMP2-induced advantages of homing, residing and growing of breast cancer cells in the bone microenvironment

**Figure 5:**
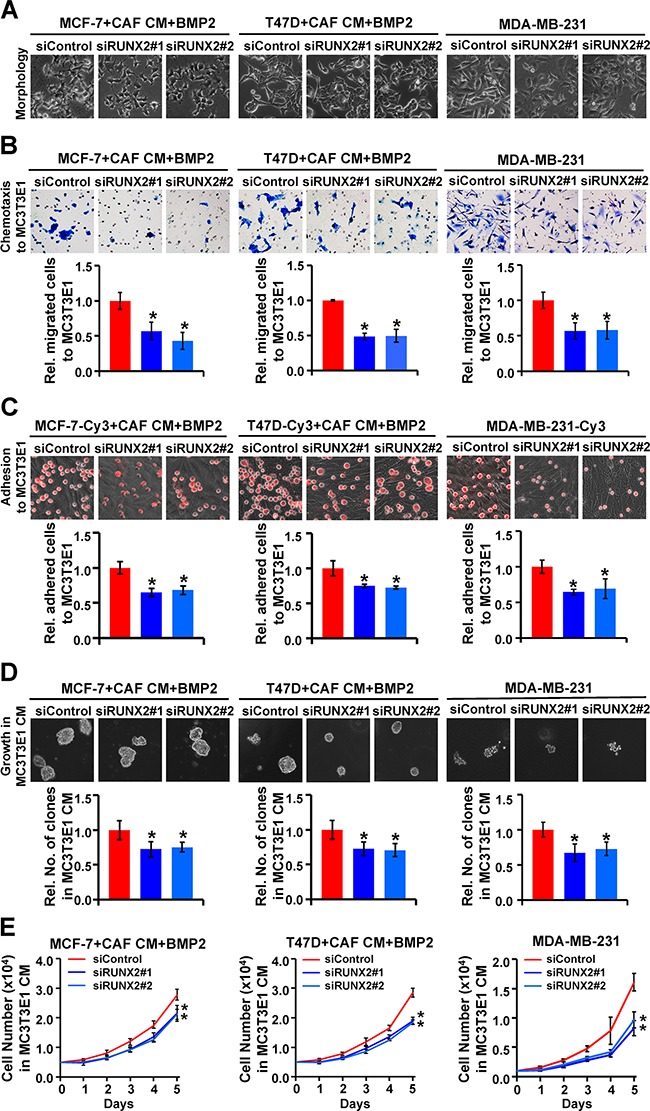
RUNX2 is critical for the CAF CM/BMP2-induced advantages of homing, residing and growing of breast cancer cells in an osteblast-mimc bone microenvironment MCF-7, T47D and MDA-MB-231 cells were treated as indicated. **A.** The morphology of cancer cells. **B.** The chemotactic migration of cancer cells towards MC3T3E1 cells was assessed by transwell assay. **C.** The adhesion of cancer cells (red, labeled with Cy3) to MC3T3E1 cells was assessed by putting cancer cells on top of MC3T3E1 cells at 100% confluence and incubating the co-culture for 30 min. **D.** The colony formation of cancer cells in soft agar with MC3T3E1 CM. Magnification: 200X. **E.** The proliferation of cancer cells in MC3T3E1 CM. Three independent experiments were performed in duplicate. The data are presented as the mean ± SD. *, *P* < 0.05 compared with the corresponding siControl.

To further investigate the role of RUNX2 during CAF/BMP2-induced advantages of homing, residing and growing of breast cancer cells in the bone microenvironment, we assessed the chemotaxis, adhesion, anchorage-independent growth and proliferation of MCF-7 and T47D cells treated as detailed as above in MC3T3E1 cell- and MG-63 cell- mimic bone microenvironment. The results revealed that RUNX2 knockdown reversed the osteoblast-like morphology (Figure 5A) and attenuated chemotactic migration (Figure 5B and Supplementary Figure S5A), adhesion (Figure 5C and Supplementary Figure S5B), anchorage-independent growth (Figure 5D and Supplementary Figure S5C) and proliferation (Figure 5E) of MCF-7 and T47D cells induced by CAF/BMP2. Consistently, RUNX2 knockdown in MDA-MB-231 cells directly suppressed chemotaxis, adhesion, anchorage-independent growth and proliferation in MC3T3E1 cell-mimic bone microenvironment (Figures 5A-5E). These results indicate that RUNX2 plays a critical role in the CAF/BMP2-induced osteomimetic transformation of epithelial breast cancer cells, which gain the advantages of tending to bone and thriving in the bone microenvironment.

### CAF/BMP2/RUNX2-induced osteomimicry enhances the multidrug resistance of breast cancer cells in the tumor and bone microenvironments

**Figure 6:**
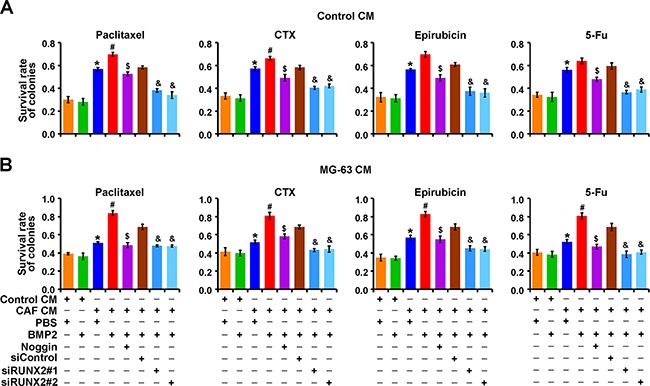
CAF/BMP2/RUNX2-induced co-expression of BRGs in breast cancer cells enhances multidrug resistance Quantification of live colonies of MCF-7 cells treated as indicated in soft agar with Control CM **A.** or MG-63 CM **B.** after drug treatment relative to untreated control cells. Three independent experiments were performed in duplicate. The data are presented as the mean ± SD. *, *P* < 0.05 compared with cells treated with Control CM plus PBS; #, *P* < 0.05 compared with cells treated with CAF CM plus PBS; $, *P* < 0.05 compared with cells treated with CAF CM plus BMP2; &, *P* < 0.05 compared with cells treated with CAF CM/BMP2 plus siControl.

Considering that the ECM, which encoded by BRGs, surrounding breast cancer cells may protect the cells from chemotherapeutic agents, we treated MCF-7 cells as described above in a 3D soft agar culture supplemented with MG-63 CM or Control CM to test the sensitivity to drugs commonly used for breast cancer treatment in clinic, including paclitaxel, cyclophosphamide (CTX), epirubicin and 5-fluorouracil (5-Fu). The results showed that the cells cultured with CAF CM showed increased drug resistance in either MG-63 CM or Control CM compared with their corresponding untreated control. Strikingly, the cells cultured with CAF CM plus BMP2 displayed a stronger resistance to cytotoxic drugs compared with those cultured with CAF CM alone in either MG-63 CM or Control CM, especially in MG-63 CM. This effect was weakened by adding noggin or silencing RUNX2 expression (Figures 6A-6B and Supplementary Figures S6A-S6B). These results indicate that RUNX2 plays a critical role in the CAF/BMP2-induced osteomimicry of breast cancer cells, which obtain multidrug resistance in both tumor and bone microenvironments, and that RUNX2 depletion efficiently abolishes these effects.

## DISCUSSION

Organ-specific metastasis depends on the intrinsic molecular characteristics of cancer cells, referred to as “pre-selected” seeds [29], and a suitable microenvironment in target organs, which is created by signals released from metastatic cancer cells, referred to as a “pre-metastatic niches” [30]. Accumulating evidence suggests that cancer cells with osteomimetic features that ectopically co-expressing BRGs have an increased bone metastatic potential [2]. In the present study, we identified a set of BRGs by analyzing co-expressed genes in primary breast cancer tissues based on our gene expression profiling dataset. The set of BRGs primarily includes genes that encode functional bone remodeling-related proteins, some of which are known to be involved in bone metastasis [2] and are included in the “bone metastasis gene signature” or “osteoblast-like gene expression signature” [31]. Importantly, we found that the set of BRGs was more highly co-expressed in breast cancer tissues compared with normal breast tissues. This result suggests that the ectopic co-expression of these BRGs is commonly existed in breast cancer tissues, which may explain why breast cancers preferentially metastasize to bone, with an incidence of bone metastasis as high as 80% in advanced breast cancer patients [32]. Moreover, we also found that these BRGs were more highly co-expressed in bone metastatic tissues than in metastatic tissues of other organs. This result supports the hypothesis that the osteomimetic feature confers on cancer cells the properties to selectively metastasize to bone.

Although it is known that the osteomimetic feature with ectopic expression of BRGs enables cancer cells to metastasize to bone, the pathogenesis of breast cancer osteomimicry remains unknown. Physiologically, the expression of BRGs initially appears during development, metabolism and the repair of damage of bone, and is derived from the directed differentiation of bone marrow mesenchymal stem cells under the induction of growth factors. BMP2 is an important growth factor for inducing the differentiation of bone marrow mesenchymal cell into osteoblasts through regulating the osteoblast-specific transcription factor RUNX2 to trigger the expression of BRGs [33]. BMP2 has been reported to be highly expressed in breast cancer tissues and is related to breast cancer bone metastasis [19, 20]. CAFs, the most abundant stromal cells in breast cancer tissues, can induce breast cancer cell EMT through paracrine TGF-β signaling [12]. Cancer cells that have undergone EMT exhibit multi-lineage differentiation potential similar to mesenchymal stem cells [16]. Therefore, we speculated that the ectopic co-expression of BRGs in breast cancers may be derived from epithelial cancer cells that have undergone EMT followed by BMP2 induction in the CAF-enriched tumor microenvironment. Indeed, by performing *in vitro* experiments that mimic the tumor microenvironment, we demonstrated that the CAF/BMP2/RUNX2 signaling axis transducted from stromal cells to cancer cells can confer breast cancer cells to obtain an osteomimetic feature that function as pre-selected bone metastatic seeds. Our results also provide insights regarding the origin of cancer tissue heterogeneity, which may arise due to stromal cell-induced EMT and stemness of cancer cells, followed by multi-lineage differentiation depending on the specific growth factor signals in the tumor microenvironment.

**Figure 7:**
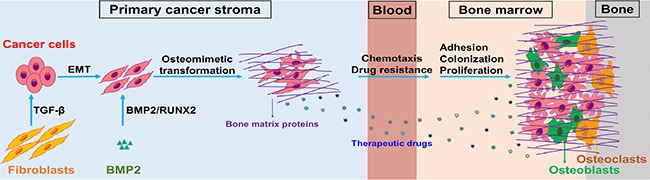
A schematic of the osteomimicry transdifferentiation of breast cancer cells CAF/BMP2 signaling in the primary cancer stroma confers an osteomimetic feature with ectopic co-expression of BRGs upon cancer cells, which acquires the advantages of chemotaxis, adhesion, colonization, proliferation and multidrug resistance in the bone microenvironment.

The formation of bone metastatic lesions is a complex process in which metastatic cancer cells interact with the bone matrix, osteoblasts, and osteoclasts to result in osteolytic or osteoblastic lesions by interrupting the balance of osteoblastogenesis and osteoclastogenesis [34]. Most of the ectopically expressed BRGs in breast cancer cells contribute to bone remodeling and bone metastasis. RUNX2, the master mediator of bone remodeling, is responsible for not only the activation of osteoblast differentiation [35] but also the regulation of osteoclast differentiation [36]. RUNX2 also contributes to the inhibition of osteoblast differentiation in breast cancer bone metastasis [37], depending on the primary tumor microenvironment and the bone microenvironment induced by metastatic cancer cells. CDH11, also known as OB-cadherin, is constitutively expressed in marrow stromal/osteoblastic cells and mediates homophilic cell adhesion [38]. CDH11 has been reported to be overexpressed in aggressive breast cancers [39] and bone metastatic breast cancer cells [8]. CDH11 selectively promotes bone metastatic homing and colonizatione of breast cancer cells and induces osteoclastogenesis through mediating interactions between breast cancer cells and marrow stromal/osteoblastic cells [40]. In both primary and metastatic cancer tissues, the ECM surrounding cancer cells not only supports cell survival, proliferation and progression, but also protects cells from chemotherapeutic agents, therefore conferring resistance to multidrug therapy [41, 42]. Our data support the hypothesis (Figure 7) that breast cancer cells with overexpression of BRGs induced by CAF/BMP2 signaling acquire the abilities of homing to, residing in and growing in the bone microenvironment, as well as resistance to multidrug therapies commonly used for breast cancer treatment in the clinic.

RUNX2 is post-translationally activated during the early stage of osteoblast differentiation to transcriptionally regulate multiple genes associated with mineralization in mature osteoblasts [43]. The activity, stability, and interactions with transcriptional co-regulators and chromatin remodeling proteins of RUNX2 under osteogenic signal stimulation are dependent on post-translational modifications, including phosphorylation, ubiquitination, and acetylation [44]. The post-translational modification of RUNX2 might be used to explain the inconsistencies that its mRNA and protein levels exhibit the same trend changes upon CAF/BMP2 induction but opposite changes upon induction with CAF alone (data not shown). RUNX2 lies downstream of the TGF-β/SMAD and BMP/SMAD signaling pathways to transcriptionally regulate the expression of various BRGs and ECM remodeling-related genes [45]. In addition to the known targets of RUNX2, including *COL1A1* [25], *COL10A1* [26] and *ITGBL1* [28], we identified *PCOLCE*, *POSTN*, *CTSK* and *ADAMTS2* as novel transcriptional targets of RUNX2 from multiple candidates in the set of BRGs. Among them, *PCOLCE* [46], *POSTN* [47] and *CTSK* [48] have been functionally validated as contributors to bone metastasis. Importantly, we found that RUNX2 differentially regulates its target genes in response to different microenvironmental stimuli: RUNX2 transcriptionally activates these genes in epithelial cancer cells that have undergone the CAF/BMP2-induced osteomimicry transformation but transcriptionally suppresses these targets in epithelial cancer cells that have undergone CAF-induced EMT, in which TGF-β/SMAD signaling induces transcriptional suppression function of RUNX2. Thus, RUNX2 functions as a master mediator during the transformation of epithelial breast cancer cells into osteomimetic cells under the induction of CAF/BMP2, and the expression of BRGs is repressed by the TGF-β/SMAD/RUNX2 signaling pathway but induced by the BMP/SMAD/RUNX2 signaling pathway. These results are consistent with the regulatory machinery of RUNX2 for skeletal gene expression [49] and the antagonism of BMP2 and TGF-β signaling during stem cell maintenance and osteogenesis [50]. Our study provides insight into the pleiotropic regulatory role of transcription factors, e.g., RUNX2 in cell fate determination and the regulation of differentiation during tissue development and tumor progression.

Indeed, this study lacks an appropriate animal model to validate our clinical and *in vitro* data due to experimental limitations. EMT process might be a transient and reversible phenomenon that is difficult to maintain especially in the complex internal environment *in vivo* [51]. In addition, we focused on EMT, not stem cells, based on the link between EMT and CSCs [14], whether the expression pattern of BRGs induced by EMT/BMP2 is similar in BMP2-stimulated CSCs will be investigated in future studies. Furthermore, besides the known target genes and those validated in this study (*PCOLCE*, *POSTN*, *CTSK* and *ADAMTS2*), multiple candidate targets of RUNX2 (Table 1) were not functionally validated in terms of their regulation by RUNX2 and contribution to metastatic bone disease. The functional elucidations of RUNX2 transcriptional targets would significantly improve the understanding of the propensity of certain cancers to metastasize to the skeleton.

In summary, our data demonstrated that the osteomimetic feature of breast cancer with ectopic co-expression of BRGs is obtained from the epithelial cancer cells that undergo stromal cell-induced EMT followed by BMP2-induced transcriptional activation function of RUNX2 in the tumor microenvironment. Our findings provide functional and mechanistic evidence to explain how advanced breast cancers commonly metastasize to bone and suggest a potential strategy for the prevention and treatment of breast cancer bone metastatic disease by targeting the EMT/BMP2/RUNX2 pathway and disrupting the interaction between cancer cells and their microenvironment.

## MATERIALS AND METHODS

### Gene expression profiling datasets

The gene expression profiling datasets of 9 normal breast cancer tissues and 49 primary breast cancer tissues were generated in our previous study [21]. The gene expression profiling datasets of 29 human breast cancer metastatic tissues, including 4 lung metastases, 15 brain metastases and 10 bone metastases, were generated by Zhang XH, et al. (GEO accession number GSE14017) [20]. GeneCards (http://www.genecards.org/) and NCBI-PubMed (http://www.ncbi.nlm.nih.gov/pubmed/) databases were used to select BRGs that encode osteoblast-specific transcription factors, osteoblast-specific adhesion molecules, bone matrix proteins, bone matrix-degrading enzymes, and growth factors that regulate bone remodeling and bone-related disease.

### Tissue specimens

Total 77 primary breast cancer tissues were collected from patients with invasive ductal carcinoma who had undergone a mastectomy at the Tianjin Medical University Cancer Institute & Hospital (TMUCIH; Tianjin, China). All tissue samples were snap-frozen in liquid nitrogen and stored at −80°C. The use of specimens in this study was approved by the Institutional Review Board of TMUCIH, and written consent was obtained from all participants.

### Cell culture and induction

The human breast cancer cell lines T47D and MDA-MD-231 were cultured in RPMI 1640 (Invitrogen, Carlsbad, CA, USA), and the human breast cancer cell line MCF-7 and the osteosarcoma cell line MG-63 were cultured in Dulbecco's modified Eagle's medium nutrient mixture basal medium (Invitrogen). The mouse preosteoblastic cell line MC3T3E1 was cultured in minimum essential medium alpha (Invitrogen). The osteoblast differentiation of MC3T3E1 cells was induced with 10 mM β-glycerol phosphate (Sigma, St. Louis, MO, USA) and 50 μg/mL L-ascorbic acid 2-phosphate (Sigma) for 14 days. Then, cancer cells were co-cultured with differentiated MC3T3E1 cells and MG-63 cells or cultured in their CM diluted with three volumes of fresh normal medium. The isolation of CAFs from primary breast cancer tissues, the collection of CM from low passage CAFs, and the EMT induction of breast cancer cells with CAF CM were performed as previously described [12]. To induce an osteomimetic phenotype of MCF-7 and T47D cells that had undergone EMT in response to CAF CM, the cells were treated with 100 ng/mL recombinant human BMP2 (Sigma) in the cultured medium for 72 h. A total of 500 ng/mL recombinant human noggin (BMP antagonist; R&D Systems, Minneapolis, MN, USA) was used to reverse the effects induced by BMP2. Phosphate-buffered saline (PBS) was added at an equal volume in a parallel test as a control. All cell cultures were supplemented with 10% fetal bovine serum (FBS; Invitrogen), 100 U/mL penicillin and 100 μg/mL streptomycin at 37°C in a humidified 5% CO_2_ incubator.

### RT-QPCR

RNA extraction from tissues or cells, reverse transcription (RT), quantitative polymerase chain reaction (QPCR) and the quantification of target gene expression were performed as previously described [52]. The sequences of primers and TaqMan probes for RT-QPCR are listed in Supplementary Table S1.

### Immunoblot and immunofluorescence

Immunoblot and immunofluorescence were performed as described previously [12]. The primary and secondary antibodies used in immunoblot and/or immunofluorescence are described in Supplementary Table S2. Band intensities of immunoblot were quantified using Image-Pro Plus 6.0.

### Plasmids and small interfering RNA transfection

To silence *RUNX2* expression in CAF/BMP2-induced MCF-7 and T47D cells or MDA-MB-231 cells, three small interfering RNAs (siRNAs) targeting unique *RUNX2* mRNA sequences (siRUNX2) were synthesized (RiboBio Co., Guangzhou, China). Two of the three siRUNX2 with optimal knockdown efficiency as determined by RT-QPCR and immunoblot were selected for the transient *RUNX2* knockdown experiments. Non-targeting siRNA (siControl) was used as a control. To force RUNX2 expression in MCF-7 and T47D cells, pcDNA3.1-RUNX2-His plasmid was transiently transfected into the cells, and the empty vector was used as a control. Plasmid and siRNA transfections were performed using Lipofectamine 2000 (Invitrogen) according to the manufacturer's instructions.

### Chemotactic migration assay

Chemotactic migration of breast cancer cells towards an osteoblast-mimic bone microenvironment *in vitro* was assessed using transwell inserts (8-μm pore size; BD Biosciences, CA, USA) in a 24-well plate. MC3T3E1 or MG-63 cells were pre-seeded in the lower chamber with culture medium containing 10% FBS for 24 h to mimic the bone microenvironment. Breast cancer cells (2.5 × 10^4^ MDA-MB-231 cells or 1 × 10^5^ MCF-7 or T47D cells) in 500 μL of serum-free medium were seeded in the upper chamber. The cells were allowed to migrate for an appropriate time. The cancer cells that migrated toward the osteoblasts were counted as described previously [12].

### Heterogeneous cell-cell adhesion assay

The heterogeneous adhesion of breast cancer cells to osteoblasts *in vitro* was assessed by seeding Cy3-labeled MCF-7, T47D, or MDA-MB-231 cells on pre-inoculated MC3T3E1 or MG-63 cells at nearly 100% confluence in a 24-well plate for the appropriate time. The non-adherent cells were removed, and washed with PBS. Then, the adherent cells were fixed with 4% paraformaldehyde and imaged using a fluorescence microscope. The non-adherent cancer cells were counted, and the percentage of adherent cells was calculated as (1- non-adherent cells / total inoculated cells) × 100%.

### Cell proliferation assay

The proliferation ability of breast cancer cells in the osteoblast-mimic bone microenvironment *in vitro* was assessed by cell growth curve analysis as previously described [28].

### Soft agar colony formation assay

Anchorage-independent cell growth was assessed using a soft agar colony formation assay. A 0.6% agarose gel in serum-free normal medium was used as the bottom gel in 6-well plates. A total of 5 × 10^3^ cells were suspended in 0.3% agarose in MC3T3E1 CM or MG-63 CM with 10% FBS and placed on top of the bottom gel. The cells were cultured for 14 days and were fed twice a week with 0.5 mL of fresh CM. Cell colonies were imaged using a phase contrast microscope, and colonies greater than 50 μm in diameter were counted.

### Drug sensitivity testing

In the culture system of soft agar colony formation assay at day nine, the cells were treated with 1 μg/mL paclitaxel, 10 μg/mL epirubicin, 10 μg/mL CTX or 50 μg/mL 5-Fu for 5 days. Cell colonies were stained with 3-(4,5-dimethylthiazol-2-yl)-2,5-diphenyltetrazolium bromide (MTT; 5 mg/mL in PBS), then imaged and counted using a microscope. Cellular drug sensitivity was determined by comparing the colony number of drug-treated cells to that of non-treated control cells.

### ChIP assay

Cancer cells transfected with pcDNA3.1-RUNX2-His were lysed and sonicated until chromatin DNA was fragmented to 300 to 500 bp. RUNX2-bound DNA was immunoprecipitated with an anti-His antibody (Cell Signaling Technology, Danvers, MA, USA), and the target fragments containing OSE2 were identified by PCR amplification using the primers listed in Supplementary Table S3. IgG was used as a negative control in the immunoprecipitations, and input DNA precipitated without antibody was used as a positive control. The ChIP-PCR products were detected by agarose gel electrophoresis and quantified by integrating optical density using Image-Pro Plus 6.0.

### Dual-luciferase reporter assay

To obtain luciferase reporter constructs for candidate RUNX2 target gene promoters containing or lacking the OSE2, the promoter sequences were amplified from human genomic DNA using the primers listed in Supplementary Table S3 and subsequently inserted into the pGL3-basic vector (Promega, Madison, WI, USA) between the KpnI and XhoI restriction sites. To assess promoter activation, the cells were seeded in 24-well plates and grown to 80% confluence. RUNX2-expressing plasmids or vector controls were co-transfected with the promoter constructs and the internal control pRL-TK into the cells. Firefly and Renilla luciferase activities were measured 48 h after transfection using a Dual-Luciferase Reporter Assay System (Promega). The relative promoter activation is presented as the ratio of firefly to Renilla luciferase activity.

### Statistical analyses

Pearson's correlation analysis was used to assess the correlation of mRNA expression between genes based on the gene expression profiling datasets and RT-QPCR data of breast cancer tissues. Chi-square test, Fisher's exact test or Wilcoxon rank sum test was applied to evaluate the differences of BRG mRNA expression between normal tissues and primary breast cancer tissues or between bone metastatic tissues and metastatic tissues in other organ. All *in vitro* experimental data are presented as the mean ± standard deviation (SD). Student's *t*-test was used to compare the differences between the experimental group and the control group. *P* < 0.05 was considered significant.

## SUPPLEMENTARY FIGURES AND TABLES


