# Supplementary material for: Breast cancer cells obtain an osteomimetic feature via epithelial-mesenchymal transition that have undergone BMP2/RUNX2 signaling pathway induction
Source: Oncotarget. 2016 Oct 27;7(48):79688–705. doi: 10.18632/oncotarget.12939 (PMC5346745; doi:10.18632/oncotarget.12939)
Supplement: Supplementary file 1 [file oncotarget-07-79688-s001.pdf]

Breast cancer cells obtain an osteomimetic feature *via* epithelial-mesenchymal transition that have undergone BMP2/RUNX2 signaling pathway induction

SUPPLEMENTARY FIGURES AND TABLES

A

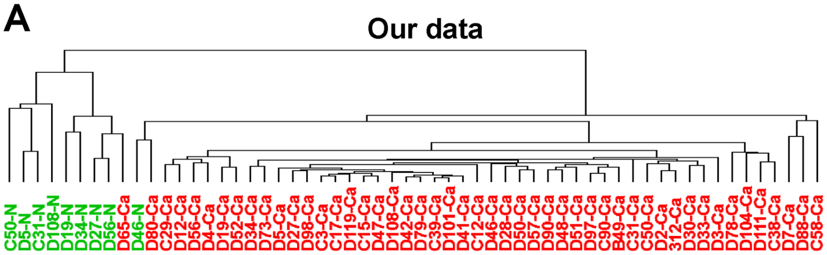

B

Grouped by BRGs expression	Tissues		*P*
	Normal	Cancer	
High	1	48	< 0.001
Low	8	1	

C

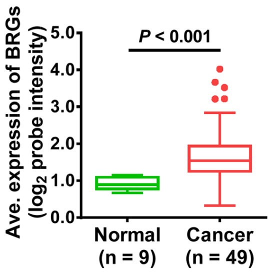

D

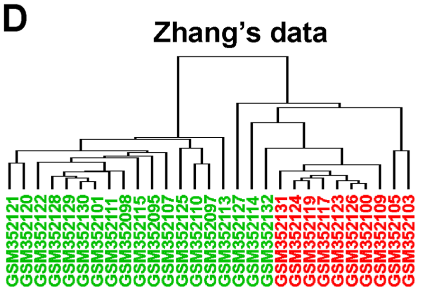

E

Grouped by BRGs expression	Metastatic tissues		*P*
	Non-bone	Bone	
High	3	10	< 0.001
Low	16	0	

F

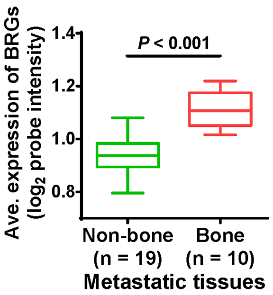

Supplementary Figure S1: Breast cancers present an osteomimetic feature with the ectopic co-expression of a set of BRGs which are prone to bone metastasis. A. Dendrograms for Figure 1B. The samples marked green are normal breast tissues (N) and the samples marked red are breast cancer tissues. **B.** The distribution of cases grouped by the expression of BRGs in (A) was analyzed using Chi-square test and Fisher's exact test. **C.** The difference of the BRGs expression levels between normal tissues and cancer tissues based on our gene expression profiling dataset was shown as Box-and-whisker plot and analyzed using Wilcoxon rank sum test. **D.** Dendrograms of Figure 1C. The samples marked green are non-bone metastatic breast cancer tissues and the samples marked red are bone metastatic breast cancer tissues. **E.** The distribution of cases grouped by the expression of BRGs in (D) was analyzed using Chi-square test and Fisher's exact test. **F.** The difference of BRGs expression levels between non-bone metastatic breast cancer tissues and bone metastatic breast cancer tissues of Zhang's gene expression profiling dataset was shown as Box-and-whisker plot and analyzed using Wilcoxon rank sum test.

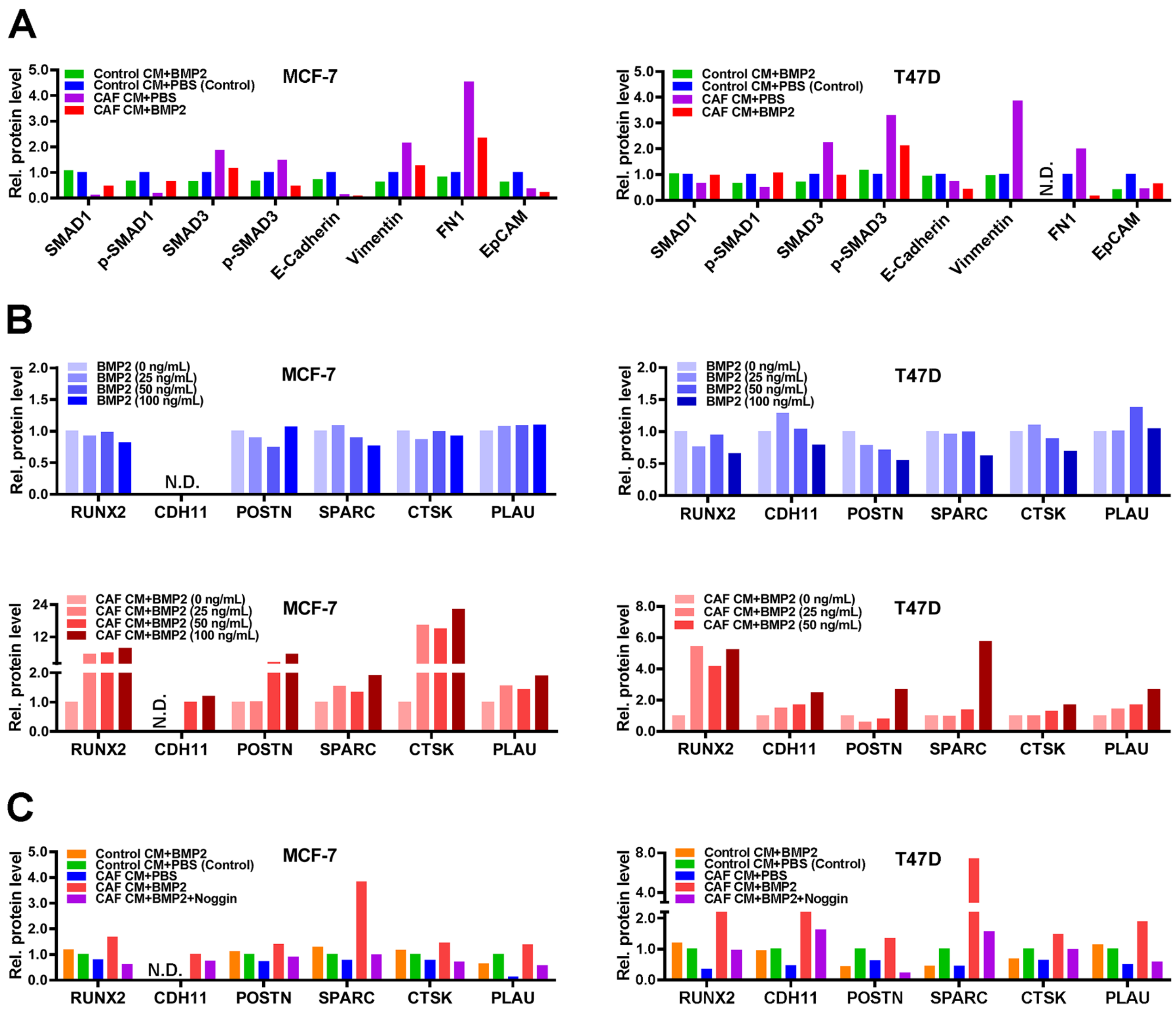

Supplementary Figure S2: The co-expression of BRGs in breast cancer cells is derived from EMT that has undergone BMP2 induction. A-C. The protein expression levels were semi-quantitatively analyzed by densitometric analysis of the immunoblot bands in Figures 2A, 2B and 2C. Data are presented as densitometric values normalized to the corresponding β-actin internal control. The relative protein expression levels are presented as the ratio of protein expression in the indicated cells compared to that in their respective control cells. N.D., not detectable.

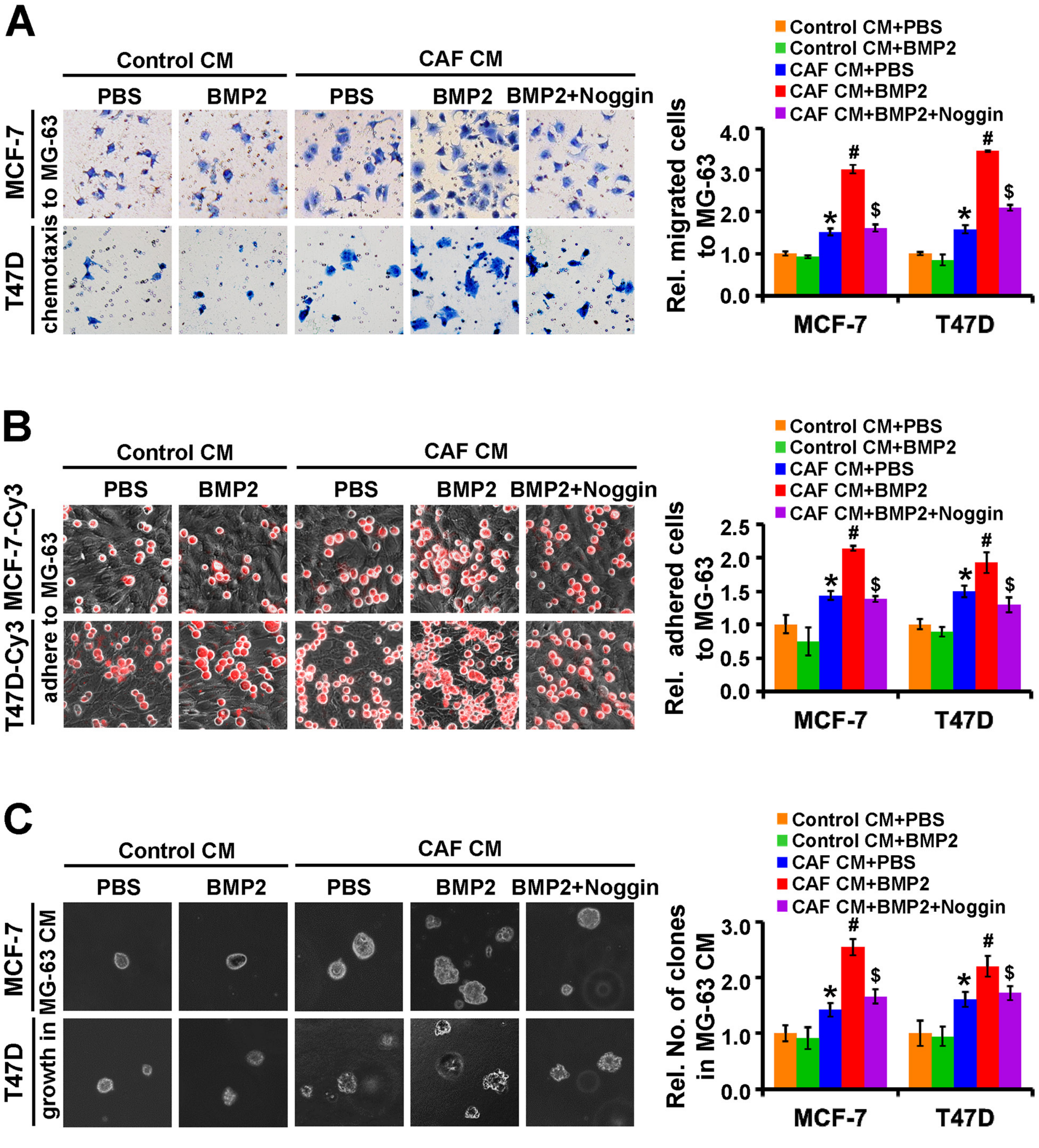

Supplementary Figure S3: The epithelial cancer cells with co-expression of BRGs induced by CAF CM/BMP2 gain the advantages of homing to, residing in and growing in the MG-63 cell-mimic bone microenvironment. MCF-7 and T47D cells were treated as indicated. **A.** The chemotactic migration of cancer cells towards MG-63 cells was assessed by transwell assays. **B.** The adhesion of cancer cells (red, labeled with Cy3) to MG-63 cells was assessed by putting cancer cells on top of MG-63 cells at 100% confluence and incubating the co-culture for 30 minutes. **C.** The colony formation of cancer cells in soft agar containing MG-63 CM. Magnification: 200X. Data are presented as the mean ± S.D. of three independent experiments performed in duplicate. *, $P < 0.05$ compared with cells treated with control CM plus PBS; #, $P < 0.05$ compared with cells treated with CAF CM plus PBS; $, $P < 0.05$ compared with cells treated with CAF CM plus BMP2.

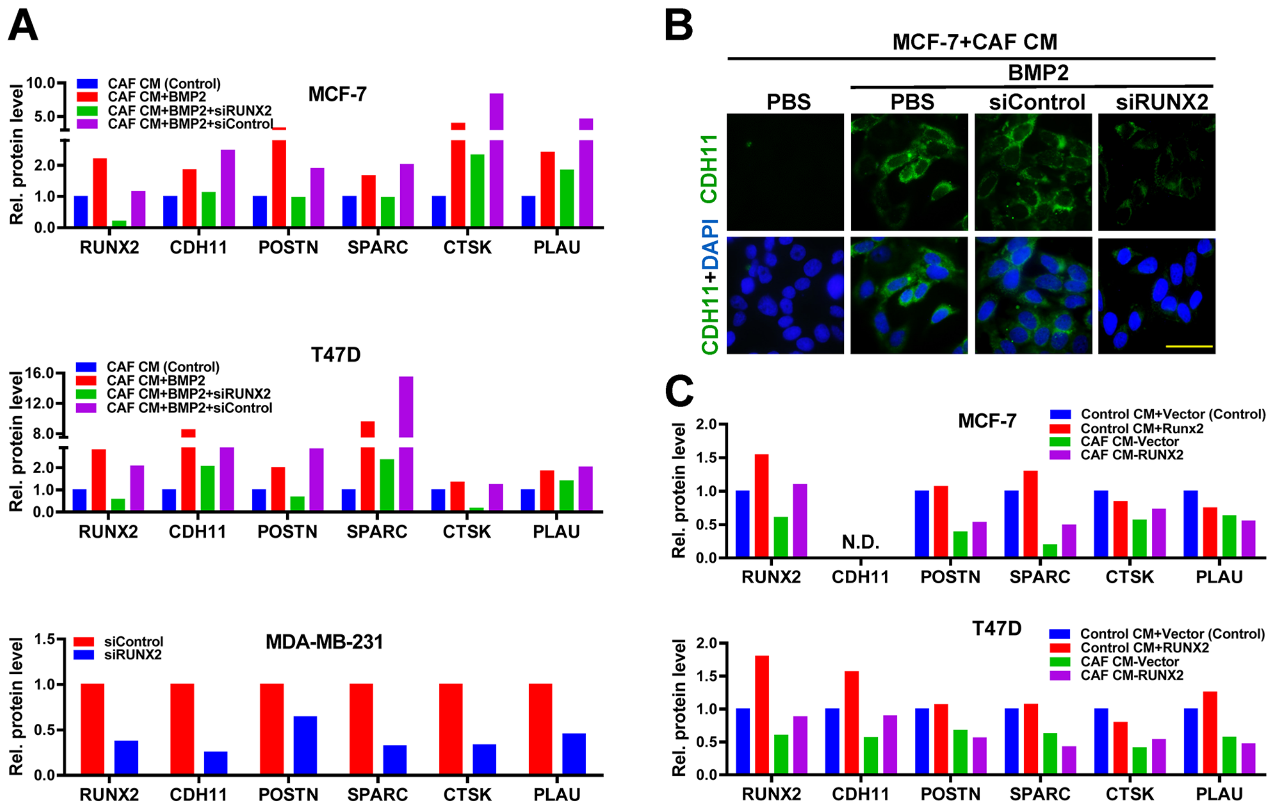

Supplementary Figure S4: RUNX2 mediates the CAF/BMP2-induced co-expression of BRGs in breast cancer cells. A. The protein expression levels were semi-quantitatively analyzed by densitometric analysis of the immunoblot bands in Figure 4A. **B.** The expression and localization of CDH11 (green) in MCF-7 cells treated as indicated were detected by immunofluorescence. DAPI staining (blue) indicates the nucleus. Scale bar, 40 μm. **C.** The protein expression levels were semi-quantitatively analyzed by densitometric analysis of the immunoblot bands in Figure 4B. Data are presented as densitometric values normalized to the corresponding β-actin internal control. The relative protein expression levels are presented as the ratio of the expression level in the indicated cells to that in their respective control cells. N.D., not detectable.

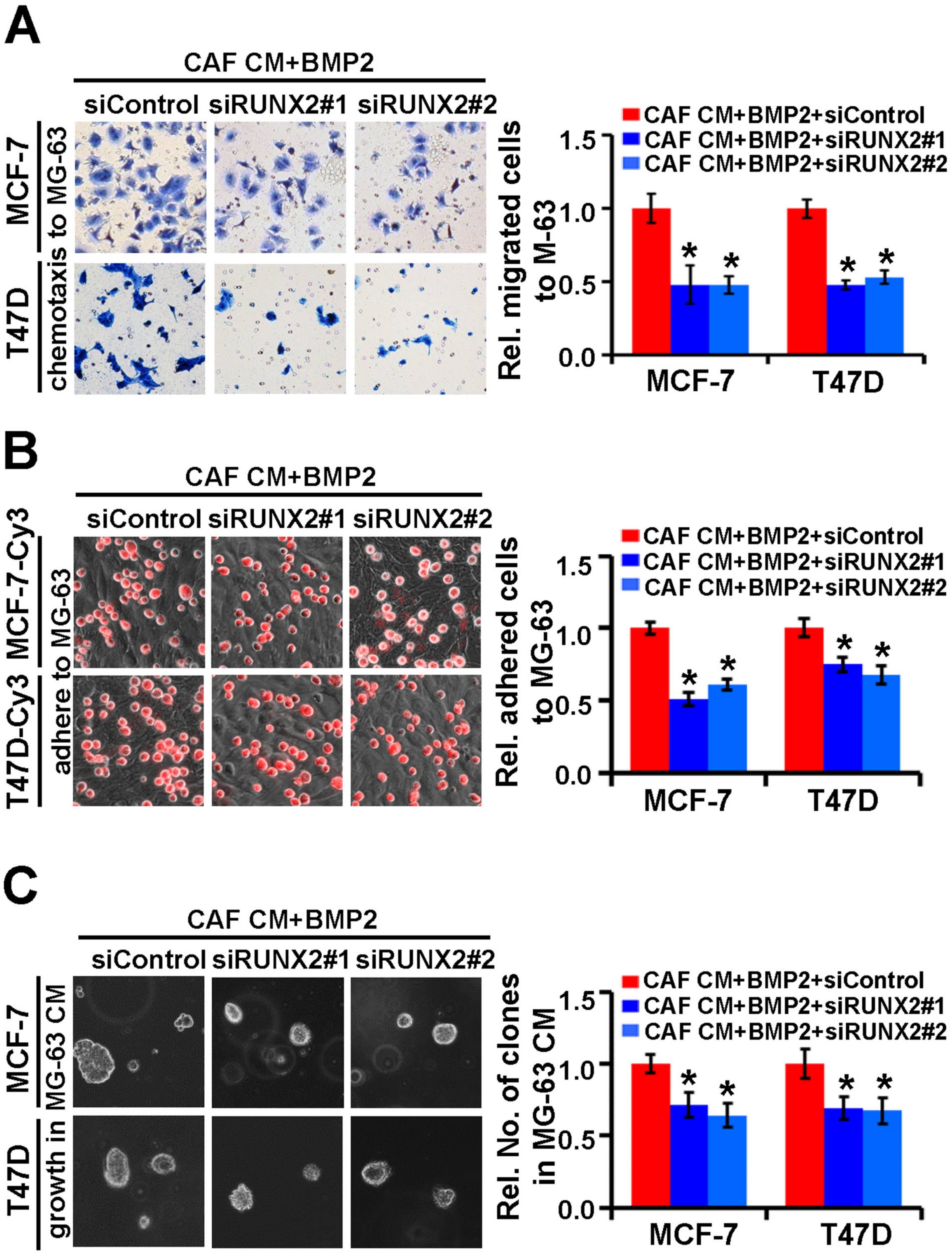

Supplementary Figure S5: RUNX2 is critical for the CAF CM/BMP2-induced ability of breast cancer cells to home to, reside in and grow in the bone microenvironment. MCF-7, T47D and MDA-MB-231 cells were treated as indicated. **A.** The chemotactic migration of cancer cells towards MG-63 cells was assessed by transwell assay. **B.** The adhesion of cancer cells (red, labeled with Cy3) to MG-63 cells was assessed by putting cancer cells on top of MG-63 cells at 100% confluence and incubating the co-culture for 30 min. **C.** The colony formation of cancer cells in soft agar with MG-63 CM. Magnification: 200X. Data are presented as the mean ± SD of two independent experiments performed in duplicate. *, $P < 0.05$ compared with the corresponding siControl.

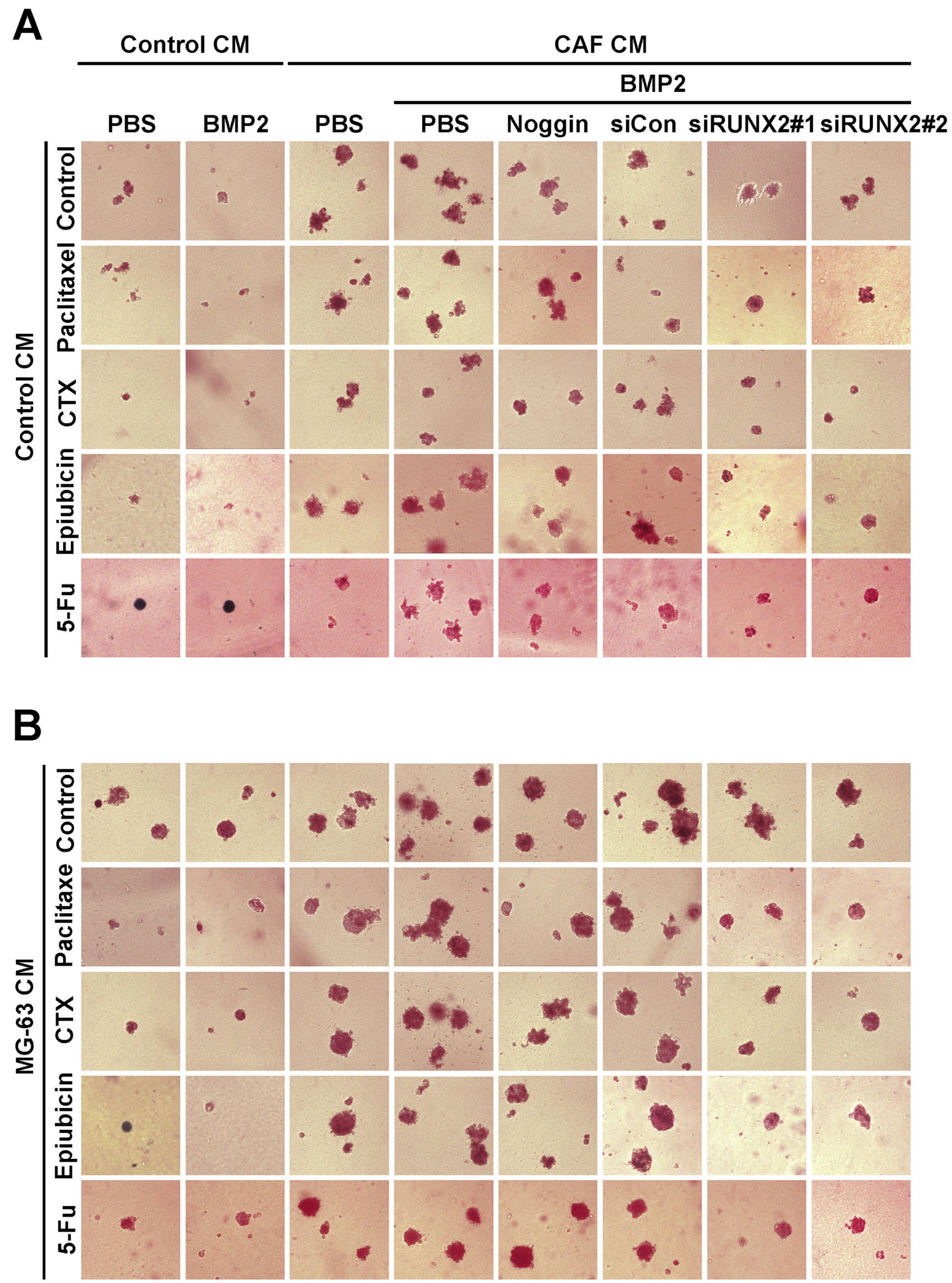

Supplementary Figure S6: CAF/BMP2/RUNX2-induced co-expression of BRGs in breast cancer cells enhances multidrug resistance. The images of live colonies of MCF-7 cells treated as indicated in soft agar with control CM **A**) or MG-63 CM **B**) after drug treatment. Live colonies were stained with MTT. Magnification: 200X.

Supplementary Table S1: Primers and TaqMan probes for RT-QPCR

Genes	Forward primers	Reverse primers	Probes (5'-FAM to 3'-TAMRA)
RUNX2	CTCTGCACCAAGTCCTTTTAATC	AGGAGGGGTAAGACTGGTCATAG	TGCCTGGGGTCTGTAATCTGAC
ITGBL1	TCGCATTGACCTTCCTCTTC	TCCCGTTGTTTTTGATATTC	CAGAGGAGCTGCTCCAGTTGATGT
SPARC	GGACAACAACCTTCTGACTGAG	CTGCCAGTGTACAGGGAAGAT	CTGCGGGTGAAGAAGATCCATGAGA
POSTN	AATGGAAGGAATGAAAGGCTG	CCTCGATCTCCTCCCTCAGT	AGCAGTTTTGCCCATTGACCATGTT

Supplementary Table S2: Antibodies used in immunoblot and/or immunofluorescence

Name	Source	Clone No.	Company
**Primary antibody**			
β-actin	Mouse	AC-15	Sigma
Vimentin	Mouse	V9	Zymed
Fibronectin 1	Mouse	P5F3	Santa Cruz
E-cadherin	Mouse	4A2C7	ZS-GB-BIO
EpCAM	Mouse	VU1D9	Cell signaling technology
Smad1	Rabbit	D59D7	Cell signaling technology
Phospho-Smad1	Rabbit	D40B7	Cell signaling technology
Smad3	Rabbit	C67H9	Cell signaling technology
Phospho-Smad3	Rabbit	C25A9	Cell signaling technology
RUNX2	Rabbit	M-70	Santa Cruz
CDH11	Rabbit	P707	Cell signaling technology
POSTN	Rabbit		Abcam
SPARC	Goat		R&D systems
CTSK	Mouse	E7	Santa Cruz
PLAU/uPA	Rabbit	H-140	Santa Cruz
**Secondary antibody**			
Anti-Mouse	Horse		Cell signaling technology
Anti-Rabbit	Goat		Cell signaling technology
Anti-Goat	Donkey		R&D systems

Supplementary Table S3: Primers for ChIP and dual-luciferase reporter assays

Genes	Region on promoters	Forward primers	Reverse primers
**Primers for ChIP assay**			
PCOLCE	–608/–359	AATTCCTTCTCCTTGGCCTA	CTTAGCCTCCCGAGTAGCTG
POSTN	–1004/–825	GATGGTGTGCAGCTTGTTTA	AGCCTTTCAATGTTACACTA
	–1344/–1137	TTCTTTTGAATTGCCCCTT	GGAACTGGACTGAACTAGAG
CTSK	–506/–298	GCTGTCATAAATAACCAGGA	AGCCTTTTATGTTCATTACCG
	–742/–599	GGGTTCTGATCACATTGCAC	CTAGGAGACTATGTTGGTTC
ADAMTS2	–1898/–1681	CGCTGTGTAAAGAAATACCCA	CATGAGTTGAATTGTGAAGGC
**Primers for dual-luciferase assay**			
PCOLCE	–226/+361	AAAATGAGCTGGCAGGGGTG	GCATAACCCCAAAGGAATCGG
	–859/+361	CCTGCCCACTAGACTTGTCC	GCATAACCCCAAAGGAATCGG
POSTN	–397/+222	TAATCAACAGCCTTTACCC	CTGATACGACTATGAGCCA
	–1235/+408	ATAAACTAAGCAGCAAGTTGT	AAAGTGCATAGATAAATCACAG
CTSK	–297/+216	AGCCTTTTATGTTCATTACCG	ATCTGTTGTCTGGCTTCGTT
	–502/+216	GCTGTCATAAATAACCAGGA	ATCTGTTGTCTGGCTTCGTT
	–1244/+216	CTAGGAGACTATGTTGGTTC	ATCTGTTGTCTGGCTTCGTT
ADAMTS2	–484/+354	CCCCTCCCCTTTCATTCCC	CGCGGAGTTTGCCCAAGTCA
	–1799/+354	GAAGAGGATGGAAGCGCTGA	CGCGGAGTTTGCCCAAGTCA
